# Does Fecal-Oral Transmission of SARS-CoV-2 Due to Low Sanitation Conditions Contribute to Low Mortality Rates From COVID-19

**DOI:** 10.7759/cureus.18557

**Published:** 2021-10-07

**Authors:** Nathan Rothschild

**Affiliations:** 1 Biotechnology, Tel-Hai College, Tel-Hai, ISR

**Keywords:** sars-cov-2, covid-19, immune response, environmental sanitation, fecal-oral infection

## Abstract

Background: The novel coronavirus disease 2019 (COVID-19) is a global pandemic generated by the severe acute respiratory syndrome coronavirus 2 (SARS-CoV-2). The primary infection site is mucosal surfaces, mainly the lungs and the intestine, where epithelial cells can be infected. COVID-19 has spread throughout the world, causing millions of deaths and hundreds of millions of confirmed infections. Despite the global spread of SARS-CoV-2, there are extreme differences between countries in mortality rates and confirmed infections.

Methods: Pearson correlations and a t-test were performed on data from 137 countries in order to test the correlation between number of deaths from diarrheal diseases (pre-COVID-19 pandemic data) as a marker for countries’ sanitation level, and the number of confirmed COVID-19 cases and deaths per million.

Results: It was found that countries’ prevalence of confirmed COVID-19 cases and deaths per million are statistically correlated with their sanitation level.

Conclusions: The hypothesis proposed in this article is that the low mortality rates from COVID-19 in countries where the level of sanitation is low are due to fecal-oral infection of the population by SARS-CoV-2, rather than infection of the respiratory system. This hypothesis is supported by the protective effect of the low sanitation level presented in this work and the fact that lung infection by SARS-CoV-2 can cause severe pathology, while infection in the intestine generally causes minor or no symptoms.

## Introduction

Coronaviruses constitute a large family of enveloped, positive-sense, single-stranded RNA viruses that cause diseases in mammals and birds [1]. There are seven coronavirus strains known to infect humans and cause respiratory diseases. Of these, four strains are endemic globally and cause only mild upper respiratory tract diseases, and three result in severe infections. These strains include the Middle East respiratory syndrome coronavirus (MERS-CoV), the severe acute respiratory syndrome coronavirus (SARS-CoV), and the severe acute respiratory syndrome coronavirus 2 (SARS-CoV-2) [2]. SARS-CoV-2 is a novel coronavirus that emerged in late 2019 in China and quickly spread throughout the world, causing the novel coronavirus disease 2019 (COVID-19). This virus has to date resulted in the confirmed infection of hundreds of millions and in the death of millions worldwide [3].

SARS-CoV-2 enters cells via attachment of its spike protein to the angiotensin-converting enzyme (ACE2), which is particularly abundant on the epithelial cells of the lungs and the small intestine. This indicates that not only the respiratory system, but also the digestive system, are potential routes of infection [4].

Respiratory symptoms, including common cold, cough, shortness of breath, runny nose and sore throat, are the most common. However, nearly half of the patients who were confirmed as having COVID-19 exhibited detectable SARS-CoV-2 RNA in their fecal samples [5]. The characteristics of gastrointestinal symptoms in COVID-19, such as lack of appetite, diarrhea, vomiting, and abdominal pain, are more insidious than the respiratory symptoms and are easily overlooked. However, some patients might have only gastrointestinal symptoms during the entire course of this disease, and some continue to shed the virus in feces, despite testing negative for respiratory samples. It has been suggested that further investigation is necessary in order to determine whether these patients represent a potentially overlooked means of transmission of SARS-CoV-2 [6].

Despite the global spread of SARS-CoV-2, mortality rates differ greatly between countries, and range from less than one death to more than 1800 deaths per million confirmed COVID-19 cases [3]. Several explanations have been suggested for these differences in mortality rates between countries, including the differences in the vitamin D level of the population [7], the age distribution of the population [8], the degree of exposure to air pollution [9], the variability in genetic polymorphisms [10-11], the vaccination policy [12], the microbiome composition [13], and cross-immunity [14]. It is, therefore, reasonable to assume that the geographical variations in COVID-19 cases are multifactorial. However, some explanations were only partially supported by statistical evidence or dealt mainly with a limited geographical area and did not examine their hypothesis globally.

Since SARS-CoV-2 can be transmitted not only through the respiratory system, but also through the fecal-oral route, it was suggested that sanitation may be an important factor in preventing the COVID-19 pandemic [15]. The COVID-19 pandemic was suggested to be more serious in developing and least developed countries struggling with the problem of ineffective waste disposal, open defecation, poor sanitation, and limited access to clean drinking water [15-16]. Surprisingly, the death toll from COVID-19 in developing and least developed countries is extremely low [17]. The hypothesis examined in this work is that a lower level of COVID-19 cases and mortality occurs in countries with a low sanitation level.

## Materials and methods

Pearson correlations (SPSS) were performed on data from 134 countries in order to test the correlation between the number of deaths from diarrheal diseases (pre-COVID-19 pandemic data) per 100,000 individuals and the number of confirmed COVID-19 cases per million. Pearson correlations were also performed in order to test the correlation between the number of deaths from diarrheal diseases per 100,000 individuals and the number of confirmed COVID-19 deaths per million. A comparative analysis between countries with less than 10 deaths and those with more than 10 deaths from diarrheal diseases per 100,000 individuals and the number of confirmed COVID-19 cases and confirmed deaths per million was conducted using a two-tailed t-test performed on mean values. COVID-19 related statistical data were extracted from Our World in Data (https://ourworldindata.org/coronavirus-source-data) according to an update from April 11, 2021. Statistical data on mortality rates from diarrhea in each country were taken from Our World in Data (https://ourworldindata.org/diarrheal-diseases) according to an update from 2018. Data for 14 countries with fewer than 600 confirmed COVID-19 cases per million were not included, in order to avoid countries in which COVID-19 did not spread in the population due to radical government intervention and avoid countries in which COVID-19 cases may not be monitored satisfactorily due to lack of test kits for SARS-CoV-2.

## Results

Analysis of data from 134 countries was performed in order to test the correlation between the number of deaths from diarrheal diseases (pre-COVID-19 pandemic data) and the number of confirmed COVID-19 cases and mortality (Table 1).

**Table 1:** List of countries and the number of deaths from diarrheal diseases (pre-COVID-19 pandemic) per 100,000 individuals (A); the number of confirmed COVID-19 cases per million (B); the number of confirmed COVID-19 deaths per million people (C).

C	B	A	Country	C	B	A	Country	C	B	A	Country
342	32899	1.1	Oman	24	2937	34.8	Ghana	65	1468	11.8	Afghanistan
70	3264	46.8	Pakistan	847	28184	0.1	Greece	803	44532	0.3	Albania
556	52122	1.1	Palestine	391	11311	21.3	Guatemala	71	2700	1.1	Algeria
1427	83058	4.6	Panama	10	1584	62.0	Guinea	17	710	98.5	Angola
8	932	74.4	Papua New Guinea	34	1869	110.9	Guinea-Bissau	1275	55698	1.3	Argentina
666	32772	3.1	Paraguay	22	1126	30.9	Haiti	1255	68225	0.5	Armenia
1658	49732	3.7	Peru	482	19741	18.1	Honduras	36	1153	0.4	Australia
135	7786	8.4	Philippines	2403	73897	1.3	Hungary	1073	63816	0.5	Austria
1537	67454	0.4	Poland	123	9680	85.5	India	383	27969	2.4	Azerbaijan
1658	81098	0.6	Portugal	155	5714	46.0	Indonesia	326	91328	1.5	Bahrain
115	65623	0.4	Qatar	765	24396	1.3	Iran	59	4123	30.0	Bangladesh
1300	52130	0.6	Romania	365	22827	1.4	Iraq	248	35438	0.1	Belarus
692	31388	0.3	Russia	969	48735	0.5	Ireland	2021	79596	2.4	Belgium
24	1802	50.1	Rwanda	727	96564	2.2	Israel	8	620	79.2	Benin
194	11422	1.8	Saudi Arabia	1884	62090	0.5	Italy	1066	24121	5.6	Bolivia
64	2351	72.1	Senegal	226	14224	1.2	Jamaica	2224	55452	0.3	Bosnia-Herzegovina
838	93977	0.5	Serbia	74	3981	0.7	Japan	270	18147	38.1	Botswana
5	10364	0.2	Singapore	755	64921	0.8	Jordan	1653	63253	3.5	Brazil
1921	67857	0.4	Slovakia	174	17089	0.5	Kazakhstan	2065	53470	0.4	Bulgaria
1978	1E+05	0.2	Slovenia	43	2700	76.9	Kenya	7	620	78.7	Burkina Faso
38	772	89.5	Somalia	329	57534	0.3	Kuwait	35	2325	62.6	Cameroon
898	26261	32.8	South Africa	233	13798	1.9	Kyrgyzstan	617	28087	2.0	Canada
34	2137	1.2	South Korea	1053	56855	0.2	Latvia	1267	55896	1.8	Chile
10	925	138.6	South Sudan	971	72469	1.2	Lebanon	1289	49500	1.8	Colombia
1633	71597	0.8	Spain	147	4998	90.0	Lesotho	25	1827	79.9	Congo
28	4429	3.7	Sri Lanka	409	24288	1.5	Libya	592	43687	2.2	Costa Rica
47	726	17.9	Sudan	1349	82764	0.3	Lithuania	10	1709	58.2	Cote d'Ivoire
1349	84897	2.3	Sweden	18	995	114.8	Madagascar	1527	70860	0.4	Croatia
1208	71354	1.0	Switzerland	59	1767	70.1	Malawi	40	7555	1.6	Cuba
78	1150	0.4	Syria	41	11096	3.3	Malaysia	311	58266	1.3	Cyprus
9	1395	15.2	Tajikistan	97	3874	55.6	Mauritania	2590	147350	1.3	Czechia
14	1443	65.8	Togo	9	936	2.3	Mauritius	421	41054	2.6	Denmark
104	5989	1.9	Trinidad-Tobago	1623	17671	4.2	Mexico	312	23708	4.3	Dominican Republic
781	22870	1.2	Tunisia	1331	59714	0.4	Moldova	979	19547	2.5	Ecuador
400	45036	0.7	Turkey	6	4326	0.9	Mongolia	121	2049	7.8	Egypt
7	899	57.8	Uganda	241	13592	3.6	Morocco	316	10097	5.5	El Salvador
880	43289	0.2	Ukraine	25	2194	69.1	Mozambique	76	5145	31.8	Equatorial Guinea
155	48728	3.4	United Arab Emirates	59	2620	21.9	Myanmar	3	972	121.5	Eritrea
1876	64562	0.8	United Kingdom	222	17837	46.2	Namibia	769	85739	0.1	Estonia
1697	94113	1.7	United States	104	9600	63.7	Nepal	27	1977	89.0	Ethiopia
407	40700	2.6	Uruguay	989	79722	1.0	Netherlands	157	14747	0.3	Finland
19	2531	0.7	Uzbekistan	27	1015	3.4	Nicaragua	1441	73395	0.7	France
62	6111	4.7	Venezuela	10	794	73.7	Nigeria	57	9272	33.4	Gabon
67	4891	78.2	Zambia	2007	67754	0.4	North Macedonia	70	2318	44.4	Gambia
103	2508	44.9	Zimbabwe	126	18995	2.7	Norway	970	72117	0.5	Georgia
.	.	.	.	24	947	50.5	Oceania	936	35919	1.7	Germany

**Figure 1:**
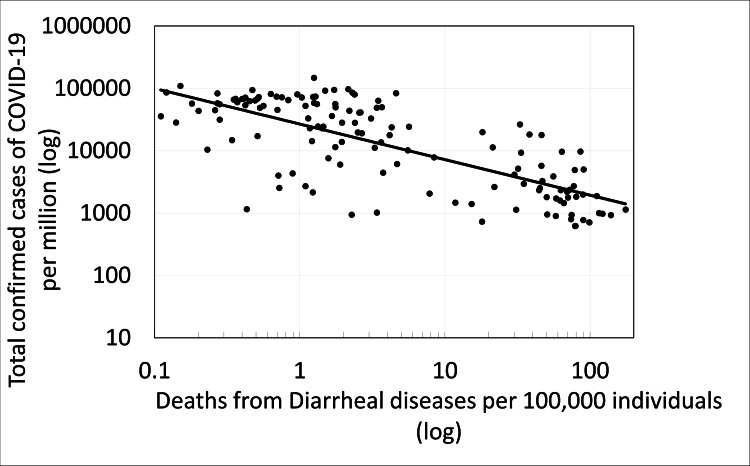
The correlation between the number of confirmed COVID-19 cases per million and the number of deaths from diarrheal diseases (pre-COVID-19 pandemic) per 100,000 individuals.

**Figure 2:**
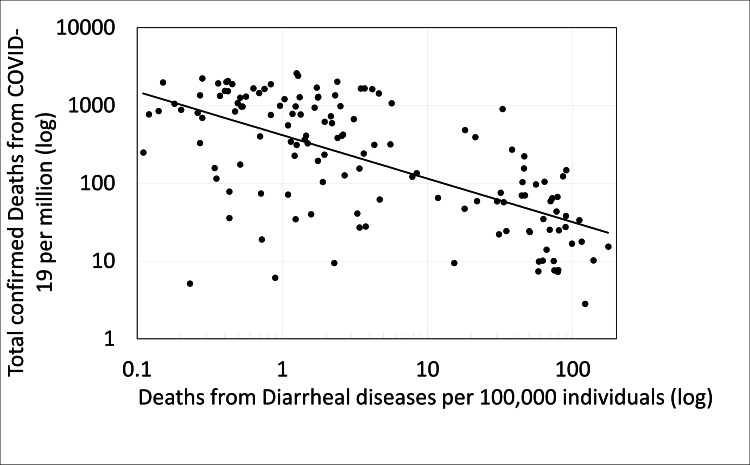
The correlation between the number of confirmed COVID-19 deaths per million and the number of deaths from diarrheal diseases (pre-COVID-19 pandemic) per 100,000 individuals.

**Figure 3:**
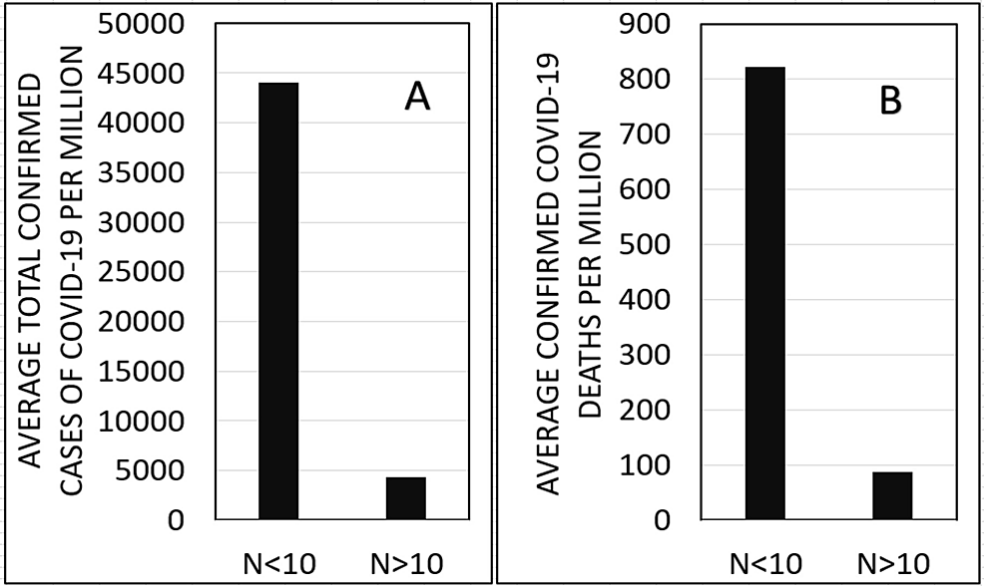
The effect of a low (N<10) and a high (N>10) number of deaths from diarrheal diseases (pre-COVID-19 pandemic) on the number of confirmed COVID-19 cases per million people (A), and on the number of confirmed COVID-19 deaths per million people (B) (N = mean number of deaths from diarrheal diseases per 100,000 individuals in the tested countries).

The findings of this work (see Figure 1) show a significant correlation between the level of sanitation in the various countries as reflected in the population’s mortality rate from diarrhea (pre-COVID-19 pandemic) and confirmed COVID-19 cases (p < 0.000, r=-0.524). In addition, a significant correlation was obtained (Figure 2) between the level of sanitation in the various countries as reflected in the population’s mortality rate from diarrhea (pre-COVID-19 pandemic) and the mortality rate from COVID-19 (p < 0.000, r=-0.471). Division of the countries into two groups according to their mortality rate from diarrheal diseases shows that the mortality rate from COVID-19 is significantly lower in countries with a low level of sanitation and a high mortality rate from diarrhea (more than 10 deaths from diarrheal diseases per 100,000 individuals) in countries with a high level of sanitation (less than 10 deaths from diarrheal diseases per 100,000 individuals) (p=3, E-13). Similar significant results were obtained when examining the effect of the mortality level from diarrheal diseases in different countries on the average number of confirmed cases of COVID-19 per million (p=7, E-21) (Figure 3).

## Discussion

In this work, it is suggested that fecal-oral infection with SARS-CoV-2 is more common under low sanitation conditions than respiratory infection, and that a high proportion of those who are infected with the virus via the fecal-oral route are asymptomatic. The wide range of COVID-19 cases and mortality rates for countries with a good level of sanitation, as evident from the very small number of deaths from diarrheal diseases, indicates that factors other than sanitation level may affect the number of COVID-19 cases and mortality rates in the absence of the protective effect of a low level of sanitation.

This hypothesis is supported by the following finding: SARS-CoV-2 can cause severe pathology in the lungs driven by an exaggerated immune response, whereas infection in the intestine generally seems to cause minor or no symptoms [5, 18-19]. Nonetheless, the results of a meta-analysis of the prevalence and mortality of COVID-19 patients with gastrointestinal symptoms indicate that the mortality among patients with gastrointestinal symptoms is similar to the overall mortality [20]. Another meta-analysis found conflicting results and reported that diarrhea is associated with increased severity of disease for COVID-19 [21]. Since this meta-analysis data was obtained from patients of countries presenting a high level of sanitation, it is likely that in most cases, the initial site of infection of COVID-19 patients was the respiratory system and that the infection of the digestive system was a secondary event and therefore may not contribute to lowering the severity of the disease. The reduced mortality reported in patients with COVID-19 who present gastrointestinal symptoms may be linked to the absence of a pro-inflammatory response in the gastrointestinal tract despite detection of SARS-CoV-2 [19]. In addition, SARS-CoV-2 neutralization is more closely correlated with IgA than IgM or IgG in the first weeks after symptom onset [22]. Furthermore, in humans, intestinal IgA are mostly dimeric, and dimeric IgA was reported to be 15 times more potent against SARS-CoV-2 than IgA monomers [23-24]. Analysis of the structural component of the corona viruses family suggested that members of this group persist in the environment for a longer period and possess the highest fecal-oral components but relatively low respiratory transmission components [25]. Indeed, immunofluorescence and polymerase chain reaction (PCR) analyses of intestinal biopsies obtained from asymptomatic individuals at four months after the onset of COVID-19 revealed the persistence of SARS-CoV-2 nucleic acids and immunoreactivity in the small intestine of 7 out of 14 individuals [26].

The well-established Syrian hamster model was recently used to experimentally delineate the relative contribution of fomite and airborne transmission and to study the impact of the SARS-CoV-2 transmission route on disease severity [27]. SARS-CoV-2 disease severity and transmission efficiency were increased for airborne compared to fomite exposure in Syrian hamsters and fomite SARS-CoV-2 exposure displayed delayed replication kinetics in the respiratory tract and led to less severe lung pathology. Since fomite exposure partially resembles the fecal-oral route of exposure in that the virus is attached to particulate material, these reports support the hypotheses that the fecal-oral route of infection by SARS-CoV-2 results in decreased severity compared to primary infection of the respiratory system.

In order to further test this hypothesis, the populations of countries suffering from a low level of sanitation as well as a low level of COVID-19 cases and mortality must be tested for the presence of SARS-CoV-2 in both feces and nasal cavity samples, in addition to determining the blood antibodies composition. This will enable increasing the population’s exposure to COVID-19, the extent to which those infected with the disease are asymptomatic, and the prevalence of the fecal-oral infection pathway. Determining the SARS-CoV-2 viral RNA sequence in these countries for individuals suffering solely from gastrointestinal symptoms will allow clarification of whether there is a mutation that improves the effectiveness of the fecal-oral infection pathway. Because the microbiome is a key part of the intestinal mucosal barrier sites and plays a major role in shaping the immune system, it may also play an important role in infection by SARS-CoV-2 and subsequent immune responses [5, 13, 28]. It is therefore suggested that the intestinal microbiome of asymptomatic and symptomatic individuals should be analyzed and compared.

## Conclusions

A new hypothesis, which can partially explain differences between countries regarding their confirmed COVID-19 cases and mortality rates, is proposed in this article. The proposed hypothesis is that the low confirmed number of COVID-19 cases and mortality rates found in countries with low levels of sanitation are due to fecal-oral infection of the population by SARS-CoV-2, rather than infection of the respiratory system.

Given that intestinal immune responses to infection with SARS-CoV-2 appear to be highly regulated, while those in the lung are exaggerated, understanding the similarities and differences between the two sites will help unravel the different immune responses in individuals suffering from mild and severe COVID-19 symptoms. This will enable better treatment of COVID-19 and the development of improved and more available vaccination techniques. This study highlights the need for developing oral vaccines which may present better immunity outcomes for the COVID-19 pandemic.
